# Willingness to use novel reversible methods of male birth control: a community-based survey of cisgender men in the United States

**DOI:** 10.1186/s40834-023-00242-y

**Published:** 2023-08-10

**Authors:** Summer L. Martins, Christy M. Boraas

**Affiliations:** 1 Allina Health, 2925 Chicago Avenue, MS 10105, Minneapolis, MN 55455 USA; 2 Department of Obstetrics, Gynecology & Women’s Health, University of Minnesota Medical School, Riverside Professional Building, 606 24th Ave. S., Minneapolis, MN 55454 USA

**Keywords:** Contraceptive attitudes, Contraceptive Behavior, Male contraception, Survey

## Abstract

**Background:**

There is high global demand for new methods of male birth control (MBC). However, contemporary evidence regarding men’s method-specific attitudes and their determinants is sparse.

**Methods:**

Non-sterilized cisgender men ages 18–45 with recent history of female sex partners were surveyed at a large community event in the Midwestern US. We examined variation in participants’ willingness to use MBC by method (gel, pill, injection, implant, and vas occlusion), potential side effects, and potential barriers. We estimated crude and adjusted prevalence ratios (aPRs) for associations between participant characteristics and willingness to use ≥ 1 MBC method.

**Results:**

Overall, 72% of participants (*n* = 187; mean age, 29) were very willing to use ≥ 1 MBC method although support for individual methods ranged widely from 62% (pill) to 24% (vas occlusion). In bivariate analysis of sociodemographic and health characteristics, few demonstrated associations with MBC willingness. In a multivariable model, willingness was independently related to age (30–39 vs. 18–29 years old, aPR = 1.24, 95% CI 1.04–1.48) and having ever been tested for HIV (aPR = 1.27, 95% CI 1.07–1.51). Willingness to tolerate side effects was < 10% for most items. The most commonly endorsed barriers to MBC use were high cost (77%) and side effects (66%).

**Conclusions:**

Enthusiasm for MBC was high but waned in the context of potential side effects and barriers. Additional research on MBC attitudes in socioeconomically and culturally diverse populations worldwide is sorely needed.

## Background

In the decades since oral contraceptives became available, reversible contraceptive options for cisgender women have proliferated to encompass a wide variety of routes, user requirements, and other attributes. Formulations aimed at the male^1^ body emerged over the same time period, targeting sperm production, function, and/or transport via hormonal and nonhormonal pathways [1]. To the frustration of researchers as well as the general public, none of these candidates have advanced to market. The pool of male birth control (MBC) methods thus remains limited to those that are permanent (vasectomy), coitally dependent (condoms), or minimally effective (withdrawal).

In anticipation of reversible MBC becoming available, studies over the past few decades have evaluated men’s attitudes toward these novel methods [2]. Clinical trials have reported participants’ acceptability of investigational MBC and their willingness to use it in the future. However, trial participants are highly selective and may not represent the broader population of potential MBC users. Studies outside of the trial setting, therefore, offer important insights into uptake of MBC but there are gaps in the existing literature. Two large global surveys of men’s willingness to use MBC were fielded ≥ 20 years ago [3, 4]. More recent evidence from the past 10 years relies on samples of students [5–7] and/or men recruited from clinical settings [7, 8]. These limitations extend to studies examining other aspects of MBC attitudes in the general population such as preferred routes, anticipated barriers and facilitators, and characteristics of people who are likely or unlikely to try MBC.

To address these evidence gaps, we conducted a cross-sectional survey of heterosexually active, cisgender men attending a large community event in the midwestern US. Our aims were to: (1) Describe men’s willingness to use MBC in general, (2) Evaluate variation in willingness according to method, potential side effects, and potential barriers, and (3) Explore a broad range of sociodemographic and health-related correlates of willingness to use MBC. By elucidating the nuances of men’s attitudes toward MBC, we hope to inform the promotion of MBC as methods advance through the development pipeline.

## Methods

### Study design and participants

We recruited participants at the 2019 Minnesota State Fair, which drew a sociodemographically diverse crowd of 2.1 million people over 12 days [9]. The infrastructure for this annual event includes a facility operated by the University of Minnesota where investigators recruit fairgoers for research studies. We promoted our study both outside the building and in more detail at the booth inside, where participants self-screened using the following inclusion criteria: (1) 18–45 years of age and (2) had penile-vaginal sex within the past five years. Eligible participants proceeded with informed consent and an anonymous, self-administered survey on an electronic tablet. Upon completion of the survey, participants received a reusable string backpack. We used REDCap tools for survey design, data collection, and data management [10]. This analysis was restricted to non-sterilized cisgender men, conceptualized as potential future users of MBC. While the survey was open to people of all genders, we excluded sperm-producing people of other gender identities due to small sample size (*n* < 5).

### Measures

**Fig. 1:**
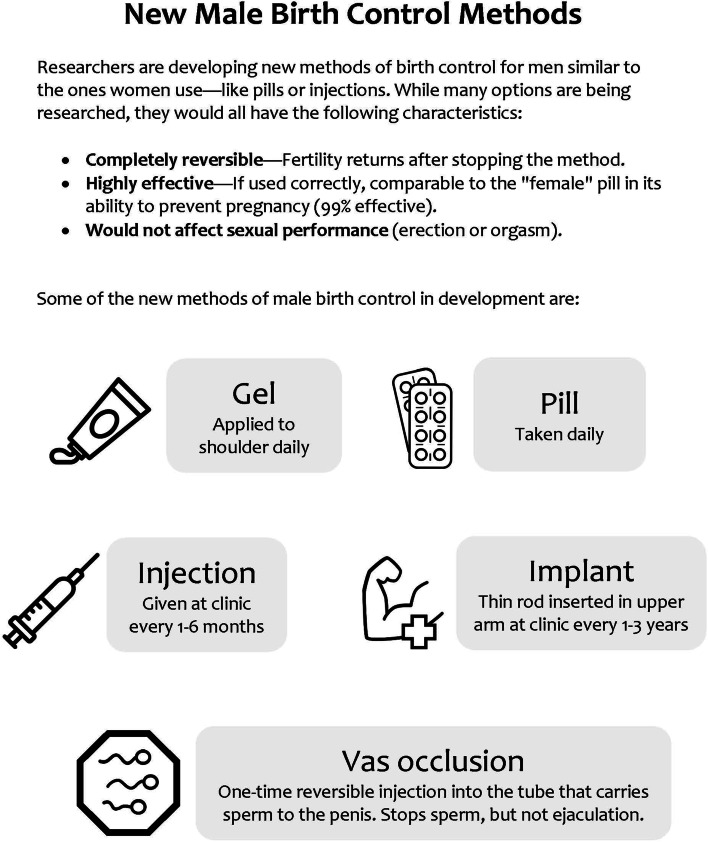
Reference sheet distributed to survey participants

Outcome measures centered on attitudes toward novel MBC methods. We gave participants an informational sheet (Fig. 1) featuring five methods—gel, pill, injection, implant, and vas occlusion—to reference while answering MBC-related questions. Participants rated their willingness to use each method on a 3-point Likert scale: very willing, somewhat willing, or not at all willing. Using the same response scale, they also reported their willingness to tolerate seven potential side effects that are both common to hormonal methods used by women and reported by participants of male contraceptive clinical trials (e.g., mood swings, weight gain) [1]. Lastly, participants were presented with a list of seven potential barriers (e.g., cost, partner disapproval) and indicated whether each would make them less willing to try MBC.

To characterize our sample and identify potential correlates of MBC willingness, we included other variables shown to be associated with contraceptive behavior in the broader literature. These included sociodemographic characteristics, relationship status, reproductive health history, religiosity, healthcare variables (insurance type, services received), mental health comorbidities, and self-efficacy. Anxiety and depression were measured using validated instruments from the U.S. National Institutes of Health’s Patient-Reported Outcomes Measurement Information System® (PROMIS) [11]. We adopted the 6-item, short-form versions of both instruments, which have exhibited high reliability and high correlation with their more comprehensive counterparts [12]. We also incorporated the short-form (4-item) General Self-Efficacy (GSE) scale from PROMIS®, hypothesizing that people with higher GSE would be more willing to try novel forms of contraception.

### Analysis

We transformed raw scores for anxiety, depression, and GSE into t-scores via the HealthMeasures Scoring Service. T-scores represent how a participant scores in relation to a reference population of U.S. adults with a mean of 50 and standard deviation of 10. We then used PROMIS®-defined thresholds to classify participants’ anxiety and depression t-scores as normal, mild, moderate, or severe and their GSE as very low, low, average, high, or very high [13].

While cleaning the data, we discovered that 32 participants (14.6% of eligible participants) confirmed that their age fell within the eligible range in screening but later skipped the survey question measuring their precise age. We suspect that the survey layout made this particular field difficult to see, as other variables had a lower percentage of missingness. Using the X^2^ statistic or Fisher’s exact test, we compared participants with known and unknown age on all correlates to determine if they were systematically different and only one variable–religious denomination–was significant (*p* < 0.05). Therefore, we excluded participants of unknown age from our analysis without major concern for selection bias.

We used univariate statistics to summarize distributions of correlates and outcome measures in the sample. Using modified Poisson regression [14], we computed unadjusted prevalence ratios (PRs) and 95% confidence intervals (CIs) to explore the relationship between each correlate and being “very willing” to use at least one MBC method. To increase available sample size in regression models, we collapsed most covariates into binary formats. Variables significant at *p* < 0.10 were entered together into a multivariable model to further examine their independent associations with willingness to use novel MBC. We conducted complete case analyses given low missingness (0.0–4.5% by variable). This study is a secondary analysis of this dataset, for which sample size was determined based on comparisons between genders. Analyses were performed using StataSE v14.2 (StataCorp LLC, College Station, Texas, USA).

## Results

Of 605 survey participants, 187 were eligible for this analysis of non-sterilized cisgender men. The sample had a mean age of 29 years (SD = 7.2) and was predominantly White/non-Hispanic (73.7%), college educated (64.5%), in a committed relationship (86.6%), and heterosexual (87.7%) (Table 1). Over half (58.7%) identified with a religious denomination, but the sample was equally split over whether religion was important in their daily lives. Mean t-scores for depression (48.4) and anxiety (49.8) indicated that distributions in our sample were similar to the general adult population (data not shown in table).

**Table 1:** Participant characteristics (*n* = 187)

Characteristic	n (%)
Age, years	
18–24	55 (29.4)
25–29	58 (31.0)
30–34	29 (15.5)
35–39	20 (10.7)
40–45	25 (13.4)
Race/ethnicity^a^	
Asian	19 (10.2)
Black or African American	6 (3.2)
Spanish, Hispanic, or Latino/Latina/Latinx	15 (8.1)
White	137 (73.7)
Multiracial	8 (4.3)
Other	1 (0.5)
Missing	1
Household income (2018)	
Less than $20,000	19 (10.2)
$20,000 to $49,999	43 (23.1)
$50,000 to $99,999	58 (31.2)
$100,000 or more	66 (35.5)
Missing	1
Highest level of education	
≤ High school diploma or equivalent	28 (15.1)
Associates degree or some college	38 (20.4)
Bachelor’s degree	70 (37.6)
Graduate or professional degree	50 (26.9)
Missing	1
Residential environment	
Urban	81 (43.3)
Suburban	86 (46.0)
Rural	20 (10.7)
Country of birth	
United States	166 (88.8)
Other	21 (11.2)
Religious denomination	
Christian, Catholic	36 (19.3)
Christian, Lutheran	27 (14.4)
Christian, Other	28 (15.0)
Other	17 (9.1)
None	79 (42.3)
Frequency of religious service attendance	
Never	77 (41.2)
Less than once per month	71 (38.0)
Once per month	16 (8.6)
Once a week or more	23 (12.3)
Importance of religion in daily life	
Not at all important	95 (50.8)
A little important	40 (21.4)
Somewhat important	26 (13.9)
Extremely important	26 (13.9)
Sexual orientation	
Straight / heterosexual	164 (87.7)
Bisexual	20 (10.7)
Gay	1 (0.5)
Prefer not to say	2 (1.1)
In committed relationship	
Yes	162 (86.6)
No	25 (13.4)
Ever involved in a pregnancy	
Yes	50 (26.9)
No	136 (73.1)
Missing	1
Ever involved in an unintended pregnancy	
Yes	15 (8.1)
No	171 (91.9)
Missing	1
Number of children in household	
0	128 (71.5)
1	22 (12.3)
2	21 (11.7)
3 or more	8 (4.5)
Missing	8
Desires pregnancy in the future	
Yes	115 (64.3)
No	64 (35.8)
Missing	8
Health insurance plan type	
Private	137 (73.3)
Public (state or federal)	29 (15.5)
Other/Multiple	9 (4.8)
None	12 (6.4)
Healthcare visit in past year for annual exam	
Yes	108 (57.8)
No	79 (42.3)
Ever been tested for HIV	
Yes	75 (40.1)
No	98 (52.4)
Unsure	14 (7.5)
Ever received at least one dose of HPV vaccine	
Yes	47 (25.1)
No	101 (54.0)
Unsure	39 (20.9)
Depression symptoms	
Normal (none)	128 (68.8)
Mild	31 (16.7)
Moderate	26 (14.0)
Severe	1 (0.5)
Missing	1
Anxiety symptoms	
Normal (none)	123 (66.5)
Mild	26 (14.1)
Moderate	31 (16.8)
Severe	5 (2.7)
Missing	2
General self-efficacy	
Very low	1 (0.5)
Low	15 (8.0)
Average	110 (58.8)
High	61 (32.6)

*HIV* Human immunodeficiency virus, *HPV* Human papillomavirus

^a^Race and Hispanic ethnicity were collected separately. For this combined variable, participants were classified first by ethnicity and then by race; thus all other categories are non-Hispanic. “Other” includes 1 participant with write-in value, “Human”

Willingness to use novel MBC varied widely by method (Table 2): 62.0% and 52.2% were very willing to use the pill and gel, respectively, but only 24.2% indicated the same for vas occlusion. Ambiguous attitudes were similar across all methods, with approximately one-third of participants indicating they were *somewhat* willing to use each one. Anticipated tolerance of MBC side effects was low overall—17% were very willing to tolerate weight gain of 5–10 pounds, but < 10% said the same for the remaining six side effects. Firmly negative attitudes were common, with > 50% of participants indicating that they would not *at all* be willing to accept mood swings, fatigue, headache, 10–20 pound weight gain, or decreased libido associated with MBC.

**Table 2:** Attitudes toward novel male birth control methods (*n* = 187)

Measure	Very willing (%)	Somewhat willing (%)	Not at all willing (%)
Willingness to use method			
Pill	62.0	28.9	9.1
Gel	52.2	34.1	13.7
Injectable	33.5	31.9	34.6
Implant	29.1	33.0	37.9
Vas occlusion	24.2	29.1	46.7
Willingness to tolerate potential side effects			
Weight gain: 5 to 10 pounds	16.6	45.5	38.0
Acne	9.1	51.6	39.3
Mood swings	9.1	31.0	59.9
Fatigue	8.6	37.4	54.0
Headaches	8.6	35.0	56.5
Weight gain: 10 to 20 pounds	7.0	24.2	68.8
Decreased libido	5.9	31.6	62.6
	%		
Less willing to use novel male birth control if…			
…it cost a lot of money	76.5		
…it caused side effects	66.3		
…partner disapproved of it	41.7		
…not sexually active	41.2		
…partner already using birth control	37.4		
…not compatible with my religious beliefs	8.0		
…no one else I know was using it	4.8		

The most commonly endorsed barriers were high cost and side effects, with 76.5% and 66.3% of participants, respectively, indicating that these factors would make them less willing to use MBC. Very few (< 10%) cited religious beliefs and lack of peer use as potential barriers.

Willingness to use at least one MBC method was high overall (72.2%) and did not significantly vary by most sociodemographic and health-related characteristics we examined in bivariate analysis (Table 3). Seven variables met our threshold of *p* < 0.10 and were included in a multivariable model. Religious denomination was also significant but was excluded from the model due to high correlation with importance of religion in daily life. Two characteristics were independently associated with being very willing to use at least one MBC method: age 30–39 vs. age 18–29 (aPR 1.24, 95% CI 1.04–1.48) and having ever been tested for HIV (aPR 1.27, 95% CI 1.07–1.51). Estimates for history of unintended pregnancy, having private health insurance, and extreme importance of religion in daily life were of borderline significance, with CIs just crossing 1.00.

**Table 3:** Correlates of being very willing to use at least one male birth control method (*n* = 187)

Characteristic	Very willing to use ≥ 1 MBC method (%)	Prevalence ratio (95% CI)	
		Unadjusted	Adjusted^a^
Age, years			
18–29	67.3	1.00 (ref.)	1.00 (ref.)
30–39	85.7	1.27 (1.07–1.51)	1.24 (1.04–1.48)
40–45	68.0	1.01 (0.75–1.36)	1.01 (0.75–1.36)
Race/ethnicity			
White, non-Hispanic	73.7	1.00 (ref.)	–
Other	67.4	0.91 (0.73–1.14)	
Annual household income (2018)			
Less than $50,000	75.8	1.00 (ref.)	–
$50,000 or more	70.2	0.93 (0.77–1.11)	
Highest level of education			
Less than college	59.5	1.00 (ref.)	1.00 (ref.)
Some college or more	75.9	1.27 (0.98–1.66)	1.20 (0.92–1.56)
Residential geography			
Urban	72.8	1.00 (ref.)	–
Suburban	72.1	0.99 (0.82–1.19)	
Rural	70.0	0.96 (0.70–1.32)	
Country of birth			
United States	72.9	1.00 (ref.)	–
Other	66.7	0.91 (0.67–1.26)	
Religious denomination			
None	77.2	1.00 (ref.)	–
Christian	64.8	0.84 (0.69–1.02)	
Other	88.2	1.14 (0.92–1.41)	
Importance of religion in daily life			
Not, a little, or somewhat important	75.2	1.00 (ref.)	1.00 (ref.)
Extremely important	53.9	0.72 (0.50–1.03)	0.70 (0.49–1.01)
Heterosexual			
No	87.0	1.00 (ref.)	1.00 (ref.)
Yes	70.1	0.81 (0.67–0.97)	0.70 (0.64–1.08)
In committed relationship			
No	72.0	1.00 (ref.)	–
Yes	72.2	1.00 (0.77–1.31)	
Ever involved in a pregnancy			
No	69.9	1.00 (ref.)	–
Yes	78.0	1.12 (0.93–1.34)	
Ever involved in an unintended pregnancy			
No	70.8	1.00 (ref.)	1.00 (ref.)
Yes	86.7	1.22 (0.98–1.53)	1.21 (0.99–1.49)
Children living in household			
No	72.7	1.00 (ref.)	–
Yes	74.5	1.03 (0.85–1.24)	
Desires pregnancy in the future			
No	78.1	1.00 (ref.)	–
Yes	68.7	0.88 (0.73–1.05)	
Health insurance plan type			
Private	75.9	1.22 (0.97–1.55)	1.23 (0.98–1.53)
Other/None	62.0	1.00 (ref.)	1.00 (ref.)
Healthcare visit in past year for annual exam			
No	68.4	1.00 (ref.)	–
Yes	75.0	1.10 (0.91–1.32)	
Ever been tested for HIV			
No/Unsure	63.4	1.00 (ref.)	1.00 (ref.)
Yes	85.3	1.35 (1.14–1.59)	1.27 (1.07–1.51)
Ever received at least one dose of HPV vaccine			
No/Unsure	72.1	1.00 (ref.)	–
Yes	72.3	1.00 (0.82–1.23)	
Any depression symptoms			
No	71.9	1.00 (ref.)	–
Yes	72.4	1.01 (0.83–1.22)	
Any anxiety symptoms			
No	72.4	1.00 (ref.)	–
Yes	71.0	0.98 (0.81–1.19)	
General self-efficacy			
Very low, low, or average	71.4	1.00 (ref.)	–
High	73.8	1.03 (0.86–1.24)	

*HIV* Human immunodeficiency virus, *HPV* Human papillomavirus, *MBC* Male birth control

^a^Estimates from multivariable model including all variables in column

## Discussion

In this study, men were generally supportive of MBC but attitudes became more nuanced in the context of formulation, side effects, and barriers. Three recent U.S.-based studies have examined men’s willingness to use MBC, all among young men. In two surveys of male undergraduate students, 35% reported high willingness to try male hormonal contraception [6] and only 29% were likely to use vas occlusion, specifically [5]. In a combined sample of students and clinic attendees aged 18–35, 45% of men were willing to use MBC [7]. These studies differ from ours in outcome measures and response scales, but support for MBC was generally higher in our sample. This discrepancy could be attributable to age, as we found that willingness to use MBC was greatest among 30–39 year-old men and this age range was not well-represented in the three prior studies. Additional evidence from the United States regarding willingness to try MBC dates from over 20 years ago (e.g., Laird [15], Heinemann et al. [3]) and is not likely to be comparable to our study. Similar limitations apply to the global evidence base beyond the United States: there is only one study published in the past 10 years, specific to a method not included in our investigation (thermal contraception [8]) and the remaining literature on men’s willingness to use MBC is concentrated in the 2000s (e.g., Heinemann et al. [3], Martin et al. [4]).

We examined variation by method and found that participants were most willing to use a pill or gel and least enthusiastic toward vas occlusion. Previous studies assessing formulation have also found a pill to be most preferable, likely due to its ease of use and men’s familiarity with the female oral contraceptive [3, 7, 16, 17]. Notably, most studies of men’s attitudes have not included gel in the list of potential options. A self-administered transdermal gel is currently the most viable method in development, with a phase-II trial underway [18]. Our findings suggest that a high percentage of men would be willing to use this new contraceptive option.

Tolerance of side effects is a key outcome in MBC clinical trials but has been underexplored in studies of the general male population. Willingness to tolerate side effects among our study participants was low, < 10% for almost all measures, and over half indicated that side effects would make them less willing to try MBC. Among women, concern over side effects has been recognized as a contributor to contraceptive non-use, dissatisfaction and discontinuation [19–21]. The gap between men’s interest in hypothetical MBC and their willingness to withstand tangible side effects warrants exploration in future research. High cost was another potential barrier cited by the majority of our participants. Some prior studies have found cost to be a salient consideration among potential MBC users [5, 22] while others [7] have not. Future work may identify the thresholds at which men consider MBC to be affordable versus cost-prohibitive.

Our study explored a wide array of sociodemographic and health-related characteristics and found that most were not associated with men’s willingness to use MBC. Only two variables, age (30–39 vs. 18–29 years) and ever-testing for HIV, were independently associated with willingness to use MBC. Reversible contraceptive methods that allow men to share the burden of pregnancy prevention with their female partners may be especially appealing to men in committed relationships who are expanding their families. Having been tested for HIV may be a proxy for high self-efficacy regarding reproductive health maintenance and care-seeking that translates to greater motivation to try MBC. Few studies have examined correlates of men’s willingness to use MBC using multivariable analysis. Similar to our findings, a newly published survey of cisgender men from the United States and Canada found no independent association between MBC willingness and education level, sexual orientation, relationship status, pregnancy history, and parenting status [23]. However, there was no effect of age (modeled continuously) and estimates were significant for abortion history and some non-White racial/ethnic subgroups. Contrary to our findings, a multi-country survey fielded in 2002 found that not desiring more children, urban residence, higher education, and higher income were significantly and independently associated with higher willingness to use MBC [3]. Age was non-significant; however, it was categorized as < 39 years vs. > 39 years. A more recent (2019) study of male U.S. undergraduate students computed adjusted estimates for willingness to pursue male hormonal contraception, but the model consisted mostly of theoretical constructs (e.g. perceived norms) and did not include the individual characteristics featured in our study [6]. Based on existing evidence, the profile of the enthusiastic MBC user remains unclear. Characteristics are likely to vary across cultural contexts, necessitating additional studies of diverse samples from multiple settings. Evidence is particularly sparse for men in non-Western countries.

Methodological strengths of this study include our recruitment from a large, community-based event and our exploration of many dimensions of MBC attitudes such as formulation preferences and potential barriers. Additionally, our survey integrated a detailed reference sheet that provided standardized definitions of MBC methods. We also note limitations. First, the study was not powered using the outcomes examined in this secondary analysis. Greater sample size would have yielded more precise estimates from our multivariable model, in which some correlates were of borderline significance. Second, we excluded 15% of eligible participants who did not provide their precise age, although there was no indication that they were systematically different from the participants without missing data. Third, our sample is comprised of cisgender men in the United States who are predominantly white and of high educational attainment. Our findings may not be generalizable to other groups of potential MBC users with different sociodemographic characteristics.

## Conclusions

New options for reversible contraception, especially those targeting sperm, are anxiously awaited by people worldwide. As these new technologies advance closer to the market, deeper analyses of the individual, interpersonal, and structural dynamics shaping uptake of sperm-focused contraception are needed. The next stages of research and development should prioritize minimizing costs and ensuring equitable access by all populations wanting to avoid unintended pregnancy.

## Data Availability

The datasets analysed during the current study are available from the corresponding author upon reasonable request.

## Abbreviations

aPR: Adjusted prevalence ratio
CI: Confidence interval
GSE: General self-efficacy
HIV: Human immunodeficiency virus
MBC: Male birth control
PR: Prevalence ratio
PROMIS: Patient-Reported Outcomes Measurement Information System®

## Footnotes

1 Throughout this paper we use ‘male’ to refer to people who produce sperm but acknowledge that not all users of MBC will identify as male.
